# Prospective analysis of time out-of-home and objectively measured walking duration during a week in a large cohort of older adults

**DOI:** 10.1186/s11556-018-0197-7

**Published:** 2018-06-16

**Authors:** Kilian Rapp, Stefanie Mikolaizak, Dietrich Rothenbacher, Michael D. Denkinger, Jochen Klenk

**Affiliations:** 1 Department of Clinical Gerontology, Robert-Bosch-Hospital, Stuttgart, Germany; 2 Institute of Epidemiology and Medical Biometry, Ulm University, Ulm, Germany; 3 Agaplesion Bethesda Clinic Ulm, Ulm, Germany

## Abstract

**Background:**

Physical activity is considered an effective measure to promote health in older people. There is evidence that the number of outdoor trips increases physical activity by increasing walking duration. The objective of this study was to analyse the relationship between daily time out-of-home and walking duration. Furthermore, predictors for walking duration and time out-of-home were evaluated.

**Methods:**

Walking duration was measured prospectively over a 1 week period by a body-fixed sensor and the time out-of-home was assessed by a questionnaire at the same days. Seven thousand, two hundred and forty-three days from 1289 older people (mean age 75.4 years) with both sensor-based measures and completed questionnaires were included in the analyses. To account for several observation days per participant multilevel regression analyses were applied. Analyses were stratified according to the time out-of-home (more or less than 100 min/day).

**Results:**

In the group with less than 100 min out-of-home, each additional minute out-of-home added 20 s to overall walking duration. If the time exceeded 100 min the additional increase of walking duration was only moderate or weak. Leaving the home once added 40 min of walking, the following trips 15 to 20 min. Increasing age, lower gait speed, comorbidities, low temperature, rain and specific week days (Sunday) decreased both the time out-of-home and walking duration. Other variables like gender (female), isolation or living with a spouse reduced the time out-of-home without affecting walking duration.

**Conclusions:**

Being out-of-home increases daily walking duration. The association is strongest if the time out-of-home is 100 min or less.

**Electronic supplementary material:**

The online version of this article (10.1186/s11556-018-0197-7) contains supplementary material, which is available to authorized users.

## Background

Physical activity (PA) has many well established positive health benefits. A large body of evidence has shown that PA reduces the risk of cardiovascular and cerebrovascular diseases, diabetes, hypertension and some forms of cancers [1, 2]. Therefore, PA is considered one of the most effective single measures to promote health during all ages [3, 4]. In older people, PA additionally reduces the risk of disability, institutionalization and death [5, 6]. However, physical inactivity, or respectively sedentary behaviour is increasing with increasing age and is particularly common in higher age-groups in high income countries [7]. Walking is the most frequently performed and one of the safest forms of PA [8]. The daily walking duration has many personal and environmental determinants. One determinant may be the time people spend outdoors or away from home. This may be particularly the case in older people for whom the time out-of-home is no longer determined by employment or work characteristics. On the other hand, older people have a variety of reasons for leaving the home which may not necessarily increase PA or walking duration as holds true for social events such as concerts, meeting friends for coffee or a visit at the physician’s office.

There is evidence from previous studies in older people that going outdoors is associated with reduced mortality [9, 10] and other beneficial health outcomes [11–14]. Therefore, a better understanding of frequency and duration of being out-of-home and the relationship with walking duration may be a basis for better informed public health decisions for older people.

Recent studies which applied accelerometers to measure PA demonstrated that outdoor time in general results in an increase in PA [15–17] which may be the mediating factor between going outdoor and the above mentioned positive health outcomes. These studies, however, did not analyse the relationship between the time out-of-home and PA in more detail. So far there is little evidence regarding the frequency of trips or the time out-of-home in relation to walking duration.

In this prospective study, we used sensor-based measures of PA from 7243 days of 1289 older people and accompanying self-reported log-book recordings to analyse the influence of the time out-of-home on daily walking duration. Furthermore, we looked at personal and environmental factors which may influence daily walking duration and the time out-of-home.

## Methods

### Study population

The ActiFE Ulm (Activity and Function in the Elderly in Ulm) study is a population-based cohort study in people aged 65 years or older, randomly selected in Ulm and adjacent regions in Southern Germany. Exclusion criteria were: being in residential care, severe cognitive impairment or serious German language difficulties. Between March 2009 and April 2010 1506 eligible individuals agreed to participate and underwent baseline assessments. The cohort and measurements taken have been previously described [18]. For the present analysis we included only people who had measurements by a body-worn accelerometer and a completed questionnaire assessing the activities out-of-home. One thousand, two hundred and eighty-nine (85.6%) participants were considered in the analyses. Every day with a 24-h sensor measurement and a completed questionnaire was included in the analyses. For more than 90% of the participants 5 or 6 days were available for analysis. In total, 7243 days with both sensor-based measures and completed questionnaires assessing the activities out-of-home were included.

All participants provided written informed consent and the Ethics Committee of the University of Ulm had approved the study (application no. 318/08 and 50/12).

### Daily walking duration

Daily walking duration was measured prospectively using a validated uni-axial accelerometer (activPAL, PAL Technologies Ltd., Glasgow, UK) [19]. The device was attached to the thigh using waterproof adhesive tape. Participants were instructed to wear the sensor over 24 h for 7 consecutive days. Only days with activity measurements over the full 24 h were considered as a valid day and included in the analysis. Accordingly, the first and the last day of the assessment period were excluded. The data processing algorithm detects upright posture as well as walking patterns and classified the activity into three categories: lying or sitting, standing and walking. Walking duration was calculated for each included day separately. Further details can be found elsewhere [20].

### Questionnaire assessing activities out-of-home

Parallel to the sensor-based measurement participants completed a questionnaire about their time periods out-of-home. The exact time of leaving and returning home was documented for each time period out-of-home. Up to four separate time periods out-of-home could be documented at each day. The total daily time out-of-home was calculated by summing up the time of all time periods out-of-home per day for each included day separately.

### Covariates

Baseline assessments were completed by trained research assistants using standardised methods. Habitual gait speed was used as a physical performance measure and assessed over a distance of 3 or 4 m depending on the conditions at the participants’ homes.

Participant’s comorbidity was assessed using the Functional Comorbidity Index, an 18-item list of diagnoses which has been shown to be stronger associated with physical function than other comorbidity scores [21]. We used a 17-item list of diagnoses since we had no information about history of angina pectoris.

The Lubben social network scale-6 has six items which assess social networks, social supports and screen for social isolation in older persons. The score was dichotomized (isolated / not isolated) according to the suggested cut-points [22].

### Statistics

Descriptive characteristics were calculated for the analysed population. In the analyses single days served as observation units instead of individual subjects. To account for the repeated measurement structure multilevel linear regression analyses were performed with the subjects on the second level and single days on the first level with either time out-of-home or walking duration as outcome parameters. Age, sex, gait speed, functional comorbidity index, marital status, education level and the Lubben social network scale were preselected as potential predictors from literature [21–24]. Age, gait speed and functional comorbidity index were included in the model as continuous variables and sex, marital status, education level and the Lubben social network as categorical variables.

Previous analyses with the same cohort have shown that walking duration was strongly associated with daily maximum temperature, daily rain and the weekday (Sunday vs. other days) [25]. Therefore, these three parameters were additionally included in the regression analyses.

The association between time out-of-home and walking duration per day was also calculated using multilevel linear regression analyses. The association is graphically presented using restricted cubic splines with knots at the 5, 35, 65, and 95% percentile, respectively. All analyses were performed using SAS version 9.4 (SAS Institute, Cary, NC, USA).

## Results

The dataset used 7243 observation days from 1289 people. The mean age of the people was 75.4 years, 43.8% were females. The mean time out-of-home was nearly 4 h per day (227 min (SD 170.9 min)) and the mean walking duration was 104 min per day (SD 49 min). On days on which people did not leave home at all (8.5% of all days) the mean (indoor) walking duration was about 50 min (Table 1). Walking duration per day increased with the daily frequency of being out-of-home. Leaving the home once, twice, 3 times or 4 times a day added 40, 60, 75 or 92 min to the participant’s daily walking duration (Table 1).

**Table 1:** Description of the study population

Included participants, N	1289
Women, N (%)	565 (43.8%)
Age in years (Mean (SD))	75.4 (6.5)
Marital status, N (%):	
Married	842 (65.4%)
Single	49 (3.8%)
Divorced/separated	85 (6.6%)
Widowed	311 (24.2%)
Education level, N (%):	
Lower education (< 10 years)	727 (57.1%)
Higher education (≥10 years)	546 (42.9%)
Functional comorbidity index (Mean (SD))	2.6 (1.7)
Gait speed in m/s (Mean (SD))	1.0 (0.3)
Lubben social network scale: isolated N (%)	332 (25.9%)
Included days, N	7243
Time out-of-home per day in minutes (Mean (SD))	227.0 (170.9)
Walking duration per day in minutes (Mean (SD))	104.0 (49.4)
Walking duration per day in minutes stratified by the frequency of leaving home per day (Mean (SD))	
- Did not leave home (Mean (SD))	49.3 (30.3)
- Once (Mean (SD))	90.3 (43.5)
- Twice (Mean (SD))	110.5 (44.0)
- 3 times (Mean (SD))	125.7 (47.7)
- 4 times (Mean (SD))	141.6 (49.6)
Daily maximum temperature in °C (Mean (SD))	12.2 (9.7)
Daily rain in mm/hour (Mean (SD))	1.9 (4.3)

*SD* standard deviation

Age had a strong effect on time out-of-home and on walking duration. An increase by 10 years reduced the time out-of-home per day by about 1 h and daily walking duration by 15 min (Table 2). Women spent 47 min less away from home than men, their daily walking duration, however, did not differ from that of men.

**Table 2:** Association between personal characteristics, outdoor temperature and rain with a) time out-of-home and b) daily walking duration

	Time out-of-home (min)		Daily walking duration (min)	
	Adjusted for age, sex β-coeff. (95%-CI)*	Mutually adjusted β-coeff. (95%-CI)*	Adjusted for age, sex β-coeff. (95%-CI)*	Mutually adjusted β-coeff. (95%-CI)*
Age (increase by 10 years)	**− 61.87 (− 71.06;-52.68)** ^†^	**− 55.23 (− 66.21; − 44.24)**	**−22.38 (− 25.55; − 19.20)** ^†^	**− 15.27 (− 19.09; − 11.45)**
Gender				
- men (reference)	Ref.	Ref.	Ref.	Ref.
- women	**−44.81 (−56.86; −32.77)** ^‡^	**− 46.95 (− 59.90; − 34.00)**	−3.56 (− 7.72; 0.60)^‡^	− 1.08 (− 5.58; 3.43)
Gait speed (increase by 0.1 m/s)	**4.00 (1.65; 6.36)**	**3.80 (1.32; 6.27)**	**1.96 (1.15; 2.77)**	**1.60 (0.74; 2.46)**
Functional comorbidity index (increase by 1 point)	**−8.13 (− 11.84; − 4.43)**	**−6.18 (− 9.90; − 2.46)**	**− 4.73 (− 5.99; − 3.48)**	**−4.01 (− 5.30; − 2.71)**
Marital status				
- Married (reference)	Ref.	Ref.	Ref.	Ref.
- Single	9.40 (− 21.95; 40.76)	30.71 (−4.51; 65.92)	− 0.10 (− 11.01; 10.80)	2.77 (−9.50; 15.04)
- Divorced/separated	**37.23 (12.88; 61.57)**	**39.37 (13.60; 65.15)**	−5.05 (− 13.51; 3.41)	− 3.87 (− 12.83; 5.09)
- Widowed	**25.35 (9.96; 40.73)**	**29.66 (14.03; 45.30)**	−3.00 (− 8.34; 2.35)	− 1.66 (− 7.10; 3.78)
Education level				
- < 10 years (reference)	Ref.	Ref.	Ref.	Ref.
- ≥10 years	4.02 (− 8.21; 16.24)	7.97 (−4.58; 20.51)	1.76 (−2.45; 5.98)	4.02 (−0.34; 8.38)
Lubben social network scale				
- not isolated (reference)	Ref.	Ref.	Ref.	Ref.
- isolated	**−21.89 (−35.58; − 8.19)**	**−18.95 (− 33.40; −4.50)**	**−5.60 (− 10.34; −0.86)**	− 2.21 (− 7.23; 2.81)
Maximum temperature (increase by 10 °C)	**31.63 (26.23; 37.03)**	**31.29 (25.51; 37.07)**	**7.45 (5.79; 9.12)**	**7.89 (6.13; 9.65)**
Rain (increase by 1 mm/h)	**− 1.65 (− 2.46; − 0.85)**	**− 2.07 (− 2.96; − 1.18)**	**−0.87 (− 1.06; − 0.68)**	**−0.96 (− 1.17; − 0.75)**
Weekday				
- Monday to Saturday	Ref.	Ref.	Ref.	Ref.
- Sunday	**−33.47 (− 41.87; − 25.08)**	**− 35.69 (− 44.88; − 26.49)**	**−13.33 (− 15.25; − 11.41)**	**− 14.75 (− 16.84; − 12.65)**

^*^β-coefficient with 95% confidence interval; significant estimates are marked in bold numbers

^†^only adjusted for sex

^‡^only adjusted for age

Gait speed was positively and comorbidity was negatively associated both with time out-of-home and walking duration. People living alone (single, divorced/separated, widowed) spent about half an hour more out-of-home each day than married people, but the marital status had no influence on walking duration.

Higher education had a non-significant positive effect on the time out-of-home and on walking duration. Participants with poorer social networks and less social support spent about 20 min less out-of-home compared to those who reported having sufficient social support. This had no influence on walking duration.

Outdoor temperature was positively and rain was negatively associated both with time out-of-home and with walking duration (Table 2).

**Fig. 1:**
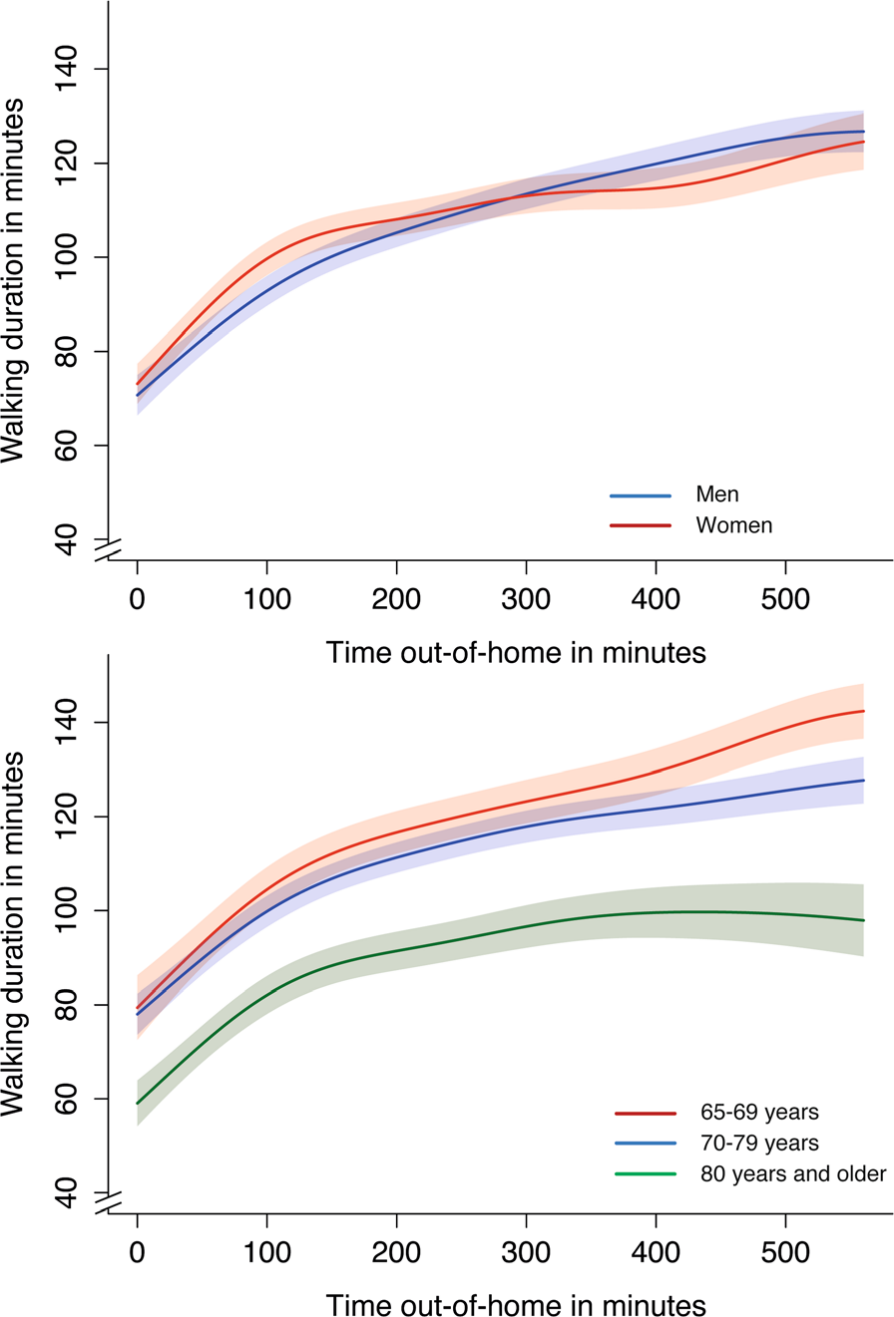
Influence of time out-of-home on walking duration stratified by sex and age (splines with 95% confidence bands)

The time out-of-home was positively associated with walking duration (Fig. 1 and Additional file 1: Table S1). The association was strongest if the time out-of-home was 100 min or less. Each minute out-of-home resulted in 20 s of additional walking duration. This was similar in women and men and there were only moderate differences between age-categories. If the time out-of-home exceeded 100 min the additional increase of the walking duration was only moderate (100–200 min) or weak (> 200 min). In contrast to younger age-categories people aged 80 years and more did not show an increase in walking duration in the time beyond 200 min out-of-home (p for interaction between the combined age-category 65–79 years and age-category ≥80 years: 0.001) (Fig. 1 and Additional file 1: Table S1).

## Discussion

Our analyses in older community-living people showed that more time out-of-home was associated with a higher daily walking duration.

This is in line with previous publications which used either outdoor time [16], the number or type of (weekly) trips [15, 26, 27] or life-space areas [17] as independent variables. We report for the first time the type of the dose-response relationship between the time out-of-home and walking duration. For those with low levels of out-of-home activity (100 min or less) each additional minute out-of-home added 20 s to the daily (indoor) walking duration. In this subgroup of people the time out-of-home is apparently dominated by activities which are associated with walking like shopping, gardening or going for a walk. If the time out-of-home exceeds 100 min, activities with no additional effect on walking duration may increasingly play a role like a visit to the physician or social contacts. Our data suggest that this may particularly be the case in people 80 years and older. Leaving the home once added 40 min of walking, the following trips 15 to 20 min. These are similar values to those reported from Davis et al. [15] who estimated an extra 20–29 min of daily walking for each trip outdoors.

The average daily time out-of-home in our study population was 3 h 47 min (227 min). It is nearly the same time as the outdoor time reported from a culturally completely different Japanese study population of the same age (3 h 37 min) [12] and somewhat less than in cohorts of older people from the US (4.2 h) [28] and Germany/Israel (4.2 h) [29]. Only few studies analysed predictors of the time out-of-home in older people [28, 29]. These studies used technical systems like an in-home activity sensor platform [30] or global positioning system (GPS) technology to assess time out-of-home. Consistently with our results, age, gender (female), social network (loneliness), rain, and weekday (Sunday) were negatively associated with the time out-of-home. In these studies low mood and depression additionally reduced the time out-of-home. In contrast to our results Petersen et al. found a higher gait speed negatively associated with the time out-of-home [28]. This is surprising and may be due to the study’s low sample size.

Interestingly, predictors for the time out-of-home do not simultaneously have to be predictors for walking duration. Women in our study, for example, spent less time out-of-home than men but their daily walking duration did not differ to that of men. This may be explainable by a still traditional division of work with women doing the domestic work like cleaning which results also in an increase of the walking duration. Participants who lived alone spent about 30 min more out-of-home than married people without accumulating more daily walking duration. It is plausible that their additional time out-of-home was mainly due to social contacts. In contrast, socially isolated people spent less time out-of-home without changing their daily walking duration. This group may be less engaged in social events which do not considerably contribute to walking duration.

It is plausible that outdoor temperature and rain influenced both outcome parameters strongly. Higher outdoor temperatures occur mainly during the summer and are therefore also associated with longer daylight. Since many older people try to avoid being out-of-home during darkness higher outdoor temperatures may also be a surrogate for a longer time window to perform activities out-of-home.

In Germany, shopping, visit of public authorities, a hairdresser or a bank is not possible on Sundays. This seems to contribute to decreased time out-of-home and the lesser walking duration on Sundays. These activities are not fully compensated by other activities typically done on a Sunday like attending a church service or going for a walk. A study with older people from the UK also observed that the least trips were undertaken on Sundays and this coincided with lower daily levels of PA [15, 26].

Strengths of our study are the large number of analysed days with information about the time out-of-home and of sensor-based measurements of the daily walking duration. There are only a few studies reporting an objective measurement of walking duration in older people [31]. Walking contributes to PA but is not identical with PA. In older people, however, walking is the most frequently performed form of PA [8] and may be therefore suitable as a surrogate of PA. Analyses with data from the ActiFE cohort demonstrated that an increase in walking duration is associated with a considerable reduction in mortality [20]. There is evidence from the literature that mainly moderate to vigorous activity has a beneficial effect on mortality [32, 33]. Walking is a composition of low, moderate (brisk walking) and vigorous (running, brisk walking up a hill) physical activity. It is a limitation of our study that we were not able to differentiate between different levels of walking intensity. Another limitation is that the questionnaire assessing activities out-of-home has not yet been validated. However, time of leaving the house and returning home is usually easy to remember, especially because participants were asked to complete the forms right after the activity has occurred. Therefore, the data should be more robust to failures as compared to detailed retrospective questionnaires of PA. Further limitations of the study are that we cannot differentiate if the time out-of-home was spent in an outdoor or an indoor environment and if the additional walking duration associated with the time out-of-home was accumulated exactly during the time out-of-home. Finally, daily habits, living conditions and environments of older people are different in different countries. This limits the external validity of our results.

Our results have implications for public health. There is no question that walking has beneficial health effects. However, a lack of motivation is a frequent barrier. To promote the number of daily trips and the total daily time out-of-home could be a measure which is rather mediated by a change of the nearby environment than by motivational procedures. Davis for example showed that an increase in amenities like the access to shops and other services within 5-min walk category increased the number of outdoor trips per week [15]. A recent publication which used a huge dataset from smartphones with built-in accelerometry demonstrated that a higher walkability of cities was associated with significantly more daily steps across all ages [34]. To build an environment which animates people to leave their home, in which shops, public authorities and services are in a walkable distance, which provides public toilets and places to rest and in which older people feel generally safe seems to be a worthwhile investment to promote public health [35]. Our results demonstrate on the other hand that the effect of the number of daily trips or the time out-of-home on walking duration is not linear and therefore limited. The type of activities may therefore play a crucial role. The latter question will be addressed in an additional publication with data from the same study population.

## Conclusions

The time out-of-home was positively associated with walking duration. The association was strongest if the time out-of-home was 100 min or less. If the time out-of-home exceeded 100 min the additional increase of walking duration was only moderate or weak. Some of the analysed variables like gender, marital status and being isolated had only an effect on the time out-of-home but not on walking duration. Our results suggest that measures which animate older people to leave their home at least once a day may increase their daily walking duration and could be therefore of value for public health.

## Additional file

Additional file 1: **Table S1.** Influence of time out-of-home on daily walking duration in all participants and stratified by sex and age. (DOCX 21 kb)
