# Supplementary material for: Prospective analysis of time out-of-home and objectively measured walking duration during a week in a large cohort of older adults
Source: Eur Rev Aging Phys Act. 2018 Jun 16;15:8. doi: 10.1186/s11556-018-0197-7 (PMC6004085; doi:10.1186/s11556-018-0197-7)
Supplement: Supplementary file 1 — Table S1. Influence of time out-of-home on daily walking duration in all participants and stratified by sex and age. (DOCX 21 kb) [file 11556_2018_197_MOESM1_ESM.docx]

**Table S1: Influence of time out-of-home on daily walking duration in all participants and stratified by sex and age**

|  | **Daily walking duration (min)*** | | | | | | | |
| --- | --- | --- | --- | --- | --- | --- | --- | --- |
|  |  |  | Sex | |  |  | Age |  |
|  | All participants  β-coeff. (95%-CI)^†^ |  | Men  β-coeff. (95%-CI)^†^ | Women  β-coeff. (95%-CI)^†^ |  | 65-69 years  β-coeff. (95%-CI)^†^ | 70-79 years  β-coeff. (95%-CI)^†^ | ≥80 years  β-coeff. (95%-CI)^†^ |
| **Time out-of-home** |  |  |  |  |  |  |  |  |
| - >0-100 min | **0.33 (0.30-0.37)** |  | **0.33 (0.29-0.38)** | **0.34 (0.29-0.38)** |  | **0.47 (0.37-0.57)** | **0.31 (0.25-0.36)** | **0.32 (0.27-0.36)** |
| - 100-200 min | **0.11 (0.05-0.16)** |  | **0.14 (0.07-0.22)** | **0.07 (-0.01-0.15)** |  | **0.05 (-0.09-0.19)** | **0.13 (0.05-0.21)** | **0.10 (0.01-0.18)** |
| - >200 min | **0.04 (0.03-0.05)** |  | **0.05 (0.04-0.06)** | **0.03 (0.01-0.05)** |  | **0.06 (0.04-0.08)** | **0.04 (0.02-0.05)** | 0.01 (-0.01-0.03) |

* adjusted for gait speed, functional comorbidity index, marital status, education level, daily maximum temperature, daily rain and weekday

^†^ β-coefficient with 95% confidence interval; significant estimates are marked in bold numbers
